# Morphological and phytochemical data of Vanilla species in Mexico

**DOI:** 10.1016/j.dib.2018.08.212

**Published:** 2018-09-07

**Authors:** Maximino Díaz-Bautista, Gabriela Francisco-Ambrosio, José Espinoza-Pérez, Hebert Jair Barrales-Cureño, Cesar Reyes, Braulio Edgar Herrera-Cabrera, Marcos Soto-Hernández

**Affiliations:** a División de Ciencias Naturales, Licenciatura en Desarrollo Sustentable, Universidad Intercultural del Estado de Puebla, Calle Principal a Lipuntahuaca S/N, Lipuntahuaca, Huehuetla, Puebla C.P. 73475, Mexico; b División de Ciencias Naturales, Ingeniería Forestal Comunitaria, Universidad Intercultural del Estado de Puebla, Calle Principal a Lipuntahuaca S/N, Lipuntahuaca, Huehuetla, Puebla C.P. 73475, Mexico; c Colegio de Postgraduados Campus Puebla, Boulevard Forjadores de Puebla No 205, San Pedro Cholula, Puebla, Mexico; d Programa en Botánica, Colegio de Postgraduados, Colegio de Postgraduados, Km. 36.5 Carr, México-Texcoco, C.P. 56230 Montecillo, Texcoco, Mexico

**Keywords:** Dendogram, Morphological data, Phylogenetic relationships, Phytochemical data, Principal component analysis, Vanilla sp

## Abstract

This systematic determination of morphological and phytochemical data was conducted with the purpose of conserving and identifying the phylogenetic relationship among the Vanilla species of the Totonacapan region in Mexico to increase awareness of the genetic biodiversity. Samples of Vanilla planifolia, V. planifolia cv. “oreja de burro”, V. pompona, V. insignis, and V. inodora, are distributed across 19 municipalities of the State of Veracruz and 19 municipalities of the State of Puebla. Morphological data parameters were determined in situ and included leaf length, leaf width, leaf thickness, stem diameter, stem thickness, node distance, stem texture degree, flower colour intensity, and fruit length. Similarly, alkaloids, tannins, saponins, phenols, flavonoids, and terpenes were determined by specifically phytochemical tests and quantified by thin layer chromatography. Both, morphological and phytochemical data parameters, were successfully used in assembling dendrograms by using the Euclidian distance method and by principal component analysis.

**Specification table**

**Table:** 

Subject area	*Botany, Taxonomy, Phytochemistry*
More specific subject area	*Genetic conservation, genetic resources and taxonomy*
Type of data	*Table, figure, graph*
How data was acquired	*Plant field collection, phytochemical assays, thin layer chromatography, morphometric measures with Vernier calliper Statistical Analysis (v. 9.0) and JMP Statistics (v10.0) software*
Data format	*Analysed*
Experimental factors	*Samples of stems and leaves from every plant were collected and then subjected to a dehydration process that required three days at a temperature of 50 °C.*
Experimental features	*An ethnobotanical exploration was conducted in order to collect morphological data from the field. Plant samples were collected for phytochemical data standard determination in the lab.*
Data source location	*There were 38 municipalities, of which 19 are located in the State of Veracruz and 19 are in the State of Puebla, Mexico. The GPS coordinates are 21° 10’ North latitude and 98° 0’ West longitude.*
Data accessibility	*The data are included in this article.*

**Value of the data**

• The collected data could increase knowledge about the level of genetic recombination among different commercial varieties with the wild population of the Vanilla genus, which are currently in danger of extinction due to excessive gathering, jointly with the asexual reproduction way.
• This data could displace some of the wild populations of Vanilla species since more than of 90% of local commercial vanillin production is extracted exclusively from Vanilla planifolia.
• In spite of a continuous debate regarding the monophyletic origin of the Vanilla genus, the morphological and phytochemical data collected could help produce an intraspecific taxonomic classification of the Vanilla genus of the Totonacapan, because of their defined and restricted geographical distributions.
• Data distribution and variability could serve as a reference for other Taxonomic studies in Mesoamerica region.

## 1 Data

The purpose of this article is to report on the collection of morphological and phytochemical data from five different vanilla species, which were successfully used in the dendrogram assembly process using the Euclidean distance method, as well as through the analysis of major components. The records relating to the morphological data indicated that the most significant differences identified were in the diameter and thickness of the stem, as well as in the distances between the knots, the degree of texture, and the intensity of the colour of the flower. As a result, the assembly dendrograms showed that V. planifolia or "oreja de burro" and V. planifolia shared the same morphological characteristics. However, they turned out to be widely different from V. pompona, V. insignis, and V. inodora. On the other hand, phytochemical data records showed that the most relevant differences found in phytochemical concentrations were in flavonoids, phenols and terpenes, in leaves and stems. In particular, the dendrogram assembled with phytochemical data showed that V. planifolia cv “oreja de burro” and V. insignis showed a phytochemical presence, which is similar to the other vanilla species tested.

The morphological and phytochemical data presented in this work addresses the opportunity to analyse phylogenetic relationships between local Vanilla species and also to preserve the genetic local background of this orchid.

**Fig. 1:**
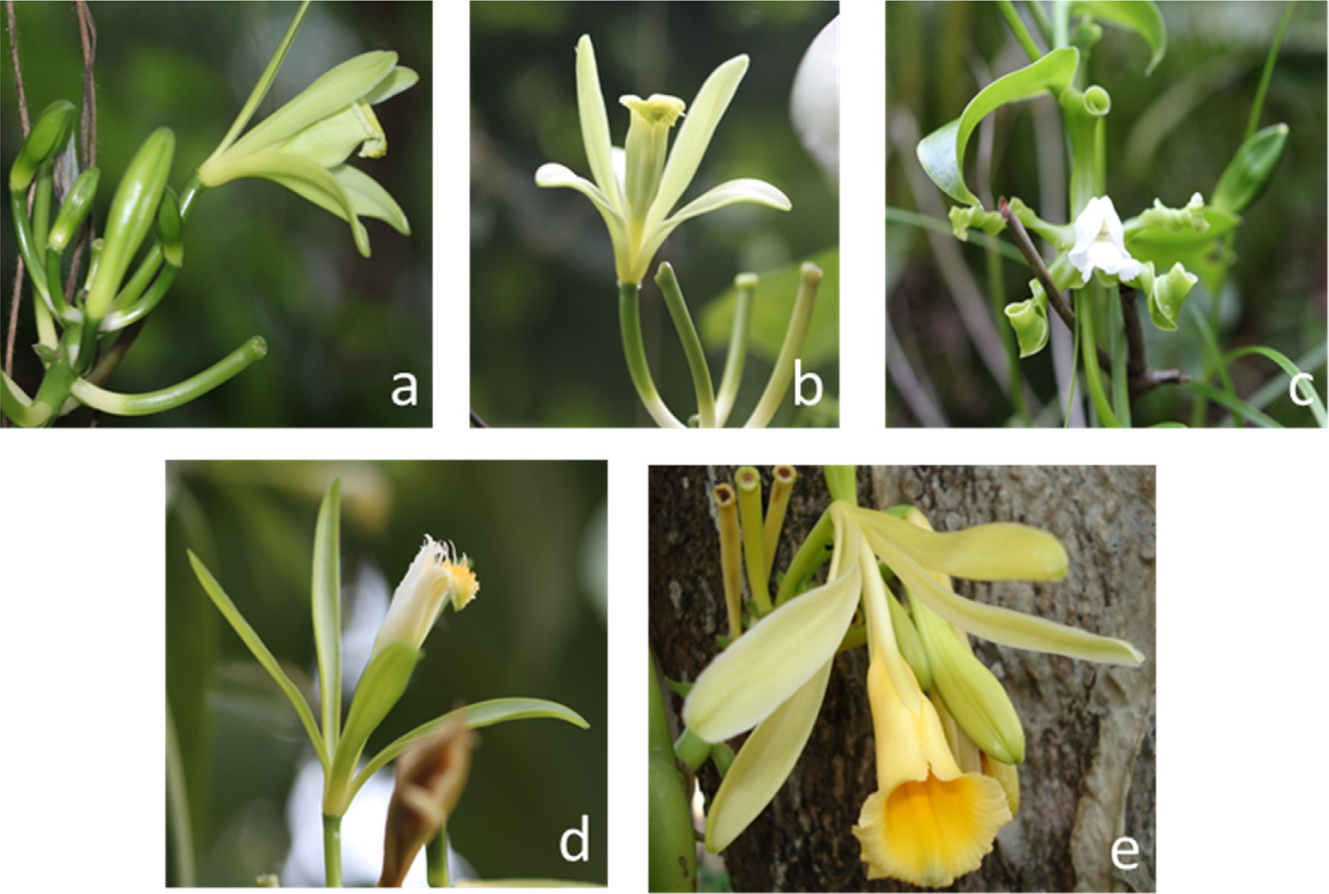
Genetic diversity of *Vanilla* genus in the Totonacapan region: a) *Vanilla planifolia*, b) *Vanilla planifolia* cv. “oreja de burro”, c) *Vanilla inodora*, d) *Vanilla insignis*, e) *Vanilla pompona*.

**Fig. 2:**
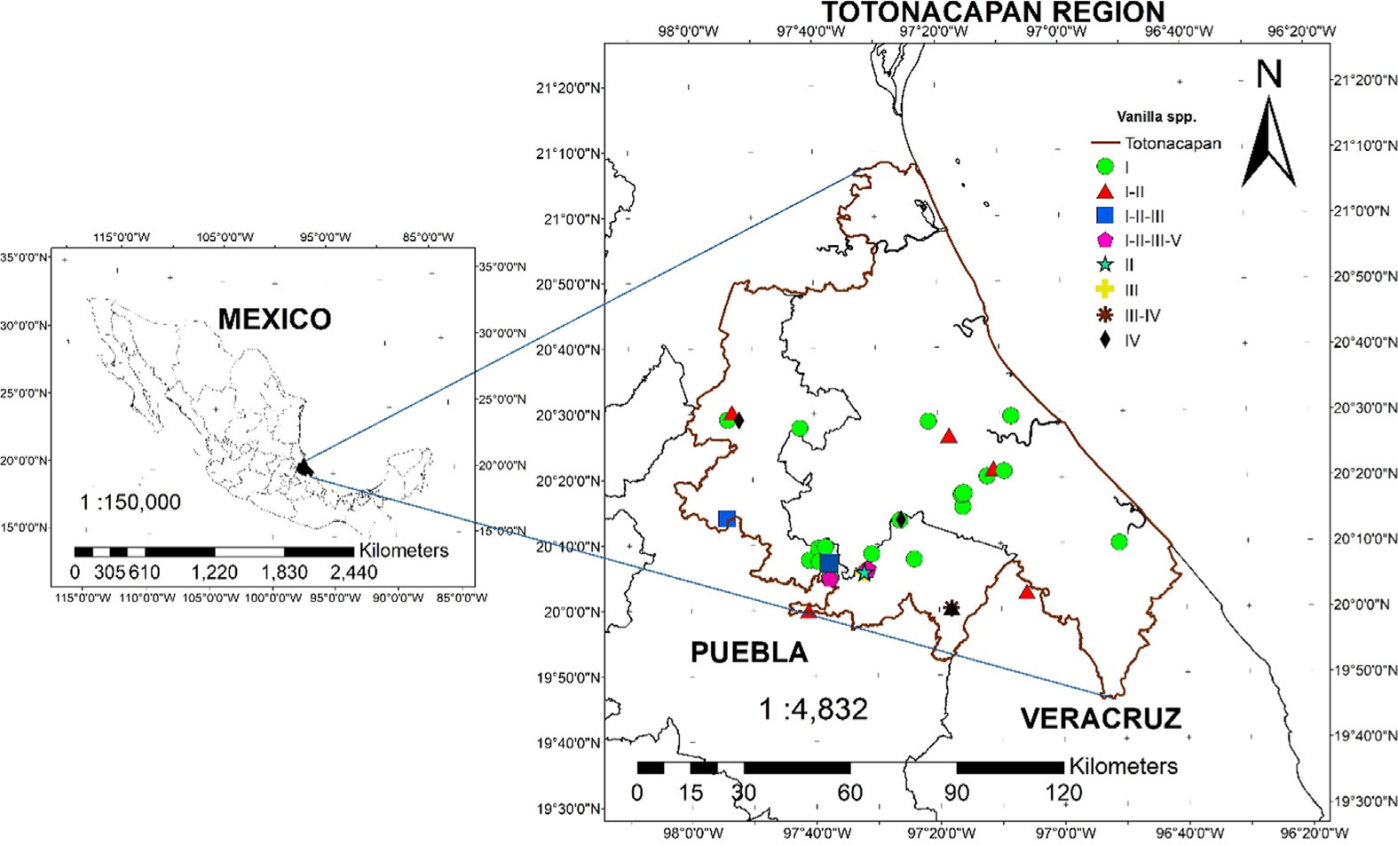
Location of the study area and distribution of *Vanilla* species. I= *V. planifolia;* II= *V. pompona;* III= *V. insignis;* IV= *V. inodora*; V= *V. planifolia* cv. *“*oreja de burro” plant.

**Fig. 3:**
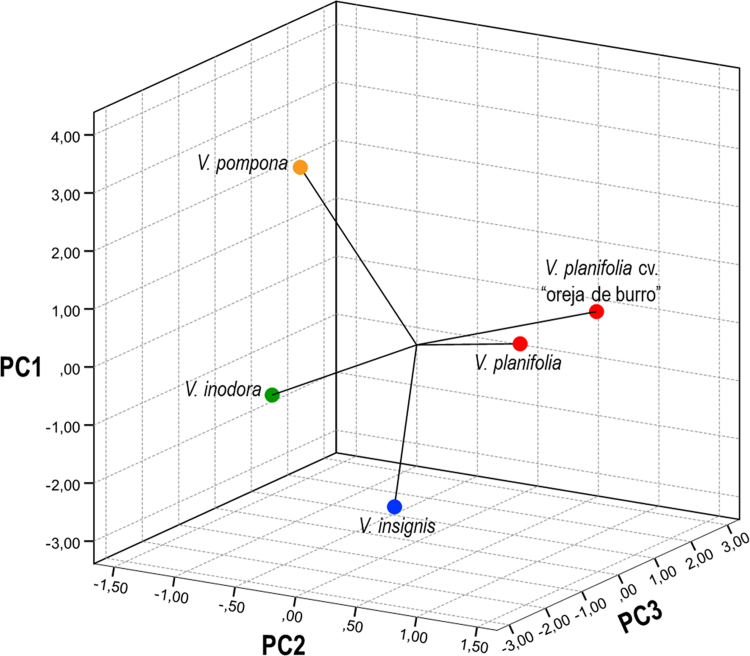
Dispersion and grouping of *Vanilla* genus based on the analysis of morphological trait data from the principal component analysis in the Totonacapan region.

**Fig. 4:**
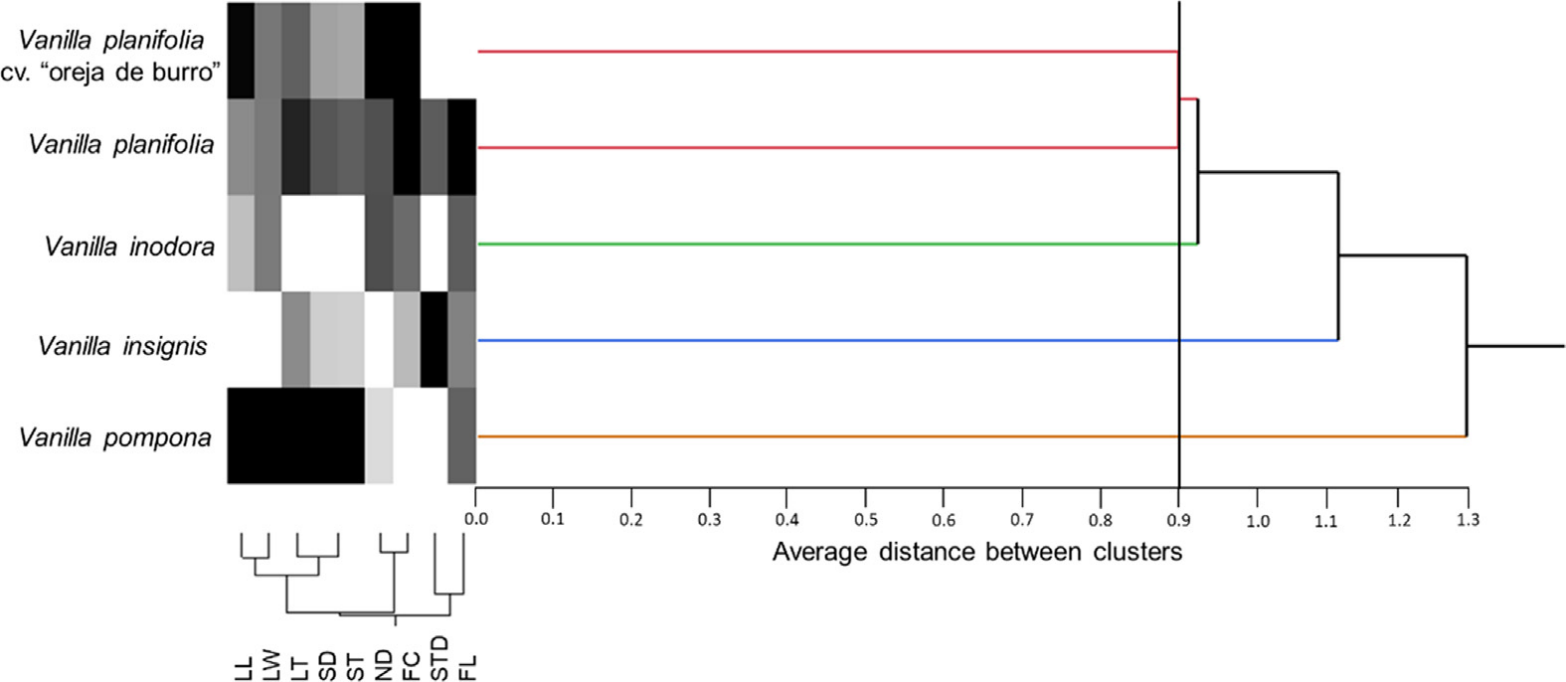
Dendrogram assembly with morphological data parameters.

Samples plant were obtained from five species of Vanilla sp. (Fig. 1*) through an ethnobotanical exploration in the Totonacapan region of Puebla-Veracruz, Mexico (*Fig. 2); in order to geo-reference their location a high-precision GPS (Garmin-650) was used in conjunction with a Universal Transverse Mercator system (TMS). Data for nine morphological traits (Table 1) were identified and the first principal components analysis explains around 89.5% of the total variation in the study (Table 2). The first principal component (PC1) explained 46.6% of the total variation and the most relevant distinctions were stem diameter (SD) and stem thickness (ST) (Table 2). The second principal component (PC2) explained 30.9% of the total variation and was determined by the distance between nodes (ND). The third principal component (PC3) explained 12% of the total variation and was obtained with stem texture degree (STD) and flower colour intensity (FC) traits (Table 2). Spatial morphological data regarding the dispersion of five vanilla species, as a function of the principal components analysis, clearly distinguished four groups of populations (Fig. 3). Assembly of the dendrogram using morphological data collected in the field is shown in Fig. 4*, where V. pompona and V. planifolia showed the most significant differences (*Table 3); the dendrogram showed the phylogenetic relationships of the five vanilla species: 1) V. planifolia and V. planifolia cv. “oreja de burro”, 2) V. inodora, 3) V. insignis, and 4) V. pompona.

**Table 1:** Medians and coefficients of variation (CV) of 9 morphological variables evaluated in *Vanilla* genus.

Traits	Species and cultivar		Median	CV
LL	101.29	***	19.87	9.81
LW	11.35	***	6.12	12.90
LT	0.02	***	0.20	14.45
SD	0.47	***	1.01	13.55
ST	5.50	***	3.22	14.32
ND	8.58	NS	11.82	18.59
STD	2.80	***	1.50	–
FC	6.40	***	3.00	–
FL	204.19	***	13.36	2.96

*** P < 0.001: NS = non-significant differences

**Table 2:** Values, eigenvectors, and cumulative rate of variation for every variable in the three first principal components in the morphological data characterisation of the *Vanilla* genus of the Totonacapan.

Variable	Code	PC1	PC2	PC3
Leaf length	LL	0.416	0.290	0.089
Leaf width	LW	0.423	0.189	−0.338
Leaf thickness	LT	0.361	−0.162	0.592^a^
Stem diameter	SD	0.462^a^	−0.166	0.122
Stem thickness	ST	0.464^a^	−0.173	0.077
Distance between Nodes	ND	−0.027	0.566^a^	0.165
Stem texture degree	STD	−0.248	−0.430	0.459^a^
Flower colour intensity	FC	−0.162	0.416	0.468^a^
Fruit length	FL	0.025	−0.345	−0.222
	Own value	4.19	2.78	1.08
	Proportion	46.6	30.9	12
	Accumulated	46.6	77.5	89.5

a Variables that represent major influence in every principal component

**Table 3:** Tukey´s range test (*p* < 0.05) performed with morphological data traits of *Vanilla* sp.

Treatment										
Trait	*V. planifolia* cv. “oreja de burro”		*V. pompona*		*V. planifolia*		*V. insignis*		*V. inodora*	
LL	21.77	b	21.60	b	17.93	bc	16.10	c	13.83	c
LW	7.70	a	5.87	b	5.82	b	5.80	b	3.41	c
LT	0.24	ab	0.20	bc	0.23	abc	0.06	d	0.17	c
SD	1.52	a	0.87	cd	1.15	bc	0.58	d	0.73	d
ST	5.07	a	2.74	bc	3.63	b	1.82	c	2.32	c
ND	10.48	a	13.77	a	12.60	a	12.62	a	9.86	a
STD	1.00	c	1.00	c	2.00	b	1.00	c	3.00	a
FC	1.00	d	4.00	a	4.00	a	3.00	b	2.00	c
FL	14.82	c	–	–	21.24	a	15.13	c	12.68	d

^*^Different letters represent the presence of significant differences

*In addition, six assays were used to determine secondary metabolite concentrations for acquiring phytochemical data (*Table 4*). Of these, only three phytochemical data assays were successfully quantified through a thin layer chromatography technique (*Table 5*).*

**Table 4:** Phytochemical screening with leaf and stem extracts of five species of *Vanilla* genus.

Treatment										
	Vob		Vpo		Vpl		Via		Vis	
	Leaf	Stem	Leaf	Stem	Leaf	Stem	Leaf	Stem	Leaf	Stem
Alkaloids	–	–	–	–	–	–	–	–	–	–
Tannins	–	–	–	–	–	–	–	–	–	–
Saponins	–	–	–	–	–	–	–	–	–	–
Phenols	+	+	+	+	+	+	+	++	+	+
Flavonoids	–	–	+	+	–	–	–	+	++	–
Terpens	–	++	+	++	+	++	–	++	–	++

Vob (*V. planifolia cv.* “oreja de burro”); Vis (*V. insignis*); Vpl (*V. planifolia*); Vpo (*V. pompona*) and Via (*V. inodora*). Concentration scale: (+) weak, (++) medium, (+++) high and (-) absence.

**Table 5:** Thin layer chromatography systems.

Secondary metabolite	Elusion system	Revealing	Comments
Phenols	Ethyl acetate-methanol: (v/v) 9:1	Derivatising agent	
Flavonoids	Ethyl acetate-methanol: (v/v): 9:1	NP-PEG reagent	
Terpenoids	Hexane-methanol 9:1; Hexane-ethyl acetate 8:2	1%Vanillin in ethanol-10% conc. sulphuric acid in ethanol	After spraying the plate, it was heated at 110 °C

**Fig. 5:**
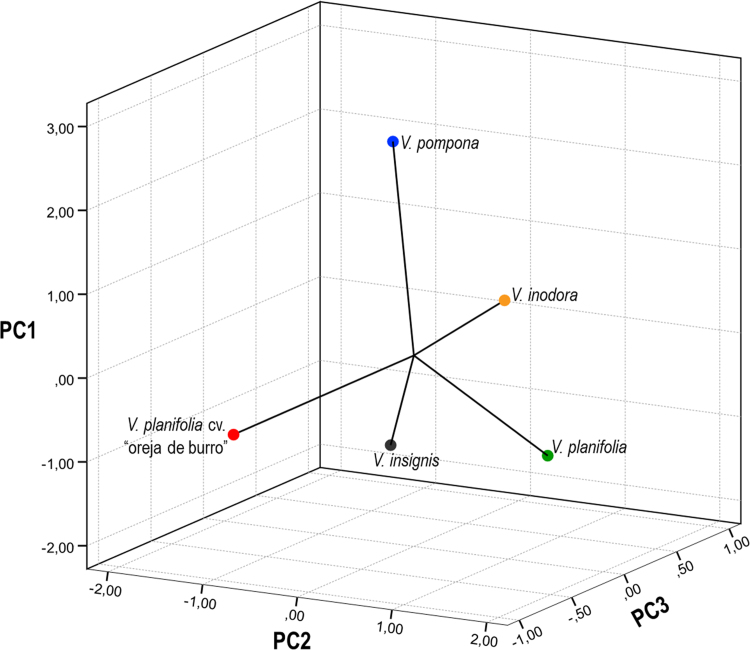
Dispersion and grouping of *Vanilla* genus based on the analysis of phytochemical trait data via principal component analysis from the Totonacapan region.

**Fig. 6:**
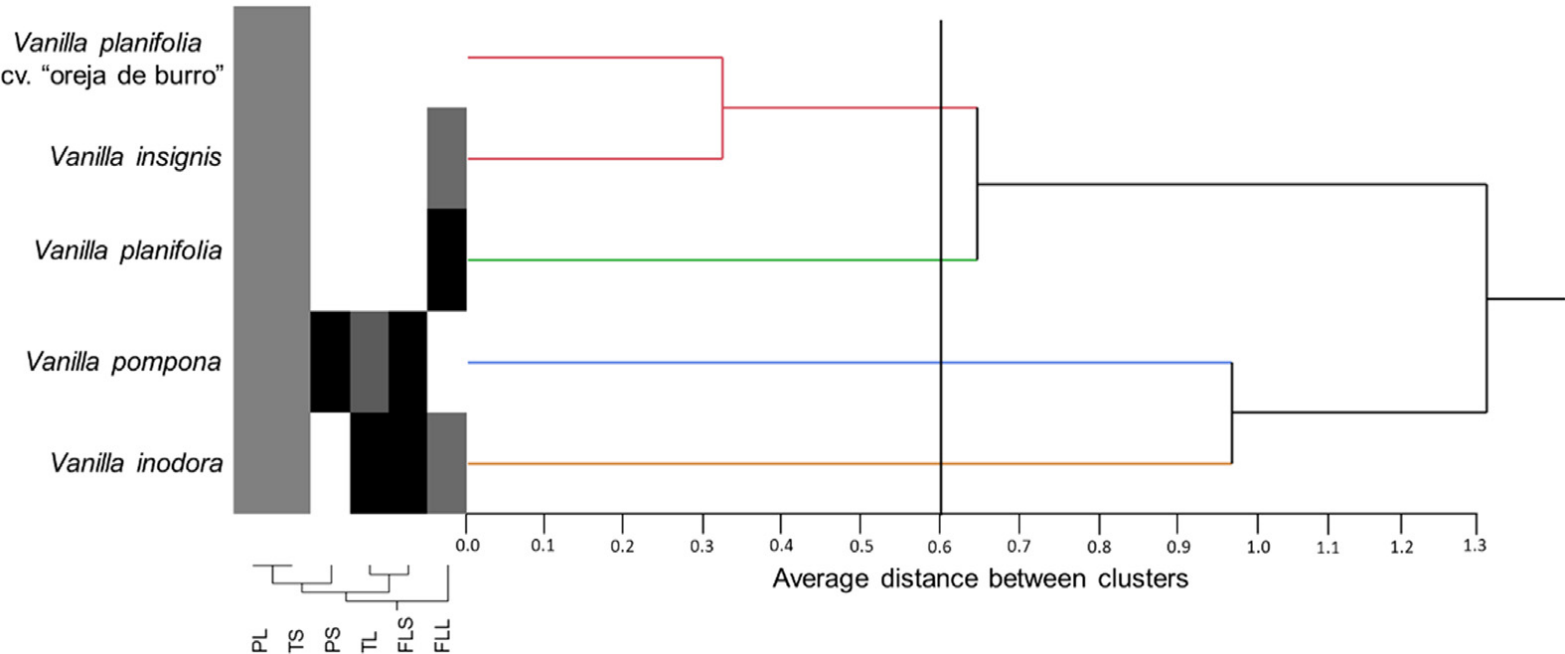
Dendrogram assembly with phytochemical data parameters.

The principal component analysis was performed using phytochemical data that only included three variables (Table 5) of six analysed. Therefore, dispersion of phytochemical data on all Vanilla species that was analysed was determined by the three first principal components which explained 92.4% of the total variation of this study (Table 6). The first principal component (PC1) explained around 51.9% of the total variation and was represented by flavonoids in stems (FS) (Table 6). PC2 explained 23.2% of the total variation and was determined by phenols in leaves (PL) and terpenes in stems (TS). The third principal component (PC3) explained only 17% of the total variation and was determined by flavonoids in leaves (PL) and terpenes in leaves (TL) (Table 6). Spatial distribution of phytochemical data on five Vanilla species is shown in Fig. 5, which was obtained by principal component analysis. Therefore, the phenols, flavonoids, and terpene concentration in leaves and stems were assembled into the dendrogram in Fig. 6. With these phytochemical data, clado analysis identified the following pattern: 1) V. planifolia cv. “oreja de burro” and V. insignis, 2) V. planifolia, 3) V. pompona and 5) V. inodora.

**Table 6:** Values, eigenvectors, and cumulative rate of variation for every variable in the three first principal components in the phytochemical data characterisation of *Vanilla* genus from the Totonacapan region.

Variable	Code	PC1	PC2	PC3
*Phenols in leaves*	PL	−0.435	0.495^a^	0.255
*Flavonoids in leaves*	FLL	−0.250	−0.495	0.507^a^
*Terpenes in leaves*	TL	0.428	0.064	0.616^a^
*Phenols in stems*	PS	0.360	0.452	−0.293
*Flavonoids in stems*	FLS	0.496^a^	0.237	0.384
*Terpenes in stems*	TS	0.435	−0.495^a^	−0.255
	Own value	3.11	1.39	1.03
	Proportion	51.9	23.2	17
	Accumulated	51.9	75.1	92.4

a Variables that represent major influence in every principal component

## 2 Experimental design, materials, and methods

### 2.1 Experimental background

In order to collect morphological and phytochemical data about the Vanilla sp, an ethnobotanical exploration was performed through in the Totonacapan region Puebla-Veracruz, Mexico; where five species of the genus Vanilla: Vanilla planifolia, V. planifolia cv. “oreja de burro”, V. pompona, V. insignis and V. inodora were identified. Phytochemical data acquisition of leaves and stems of plants was followed with a methanolic extraction method in order to quantify alkaloids, tannins, saponins, phenols, flavonoids, and terpenes (Table 4*) by using a thin layer chromatography technique. This technique identified nine morphological parameters of the plant (*Table 3). Finally, a principal component analysis was used to identify the most influential morphological and phytochemical traits impacting the data dispersion of the Vanilla species. Then a clustered analysis of phytochemical and morphological data obtained via Euclidian methods was performed in order to assemble the phylogenetic relationships.

### 2.2 Study area

The study of five species of Vanilla was conducted in Totonacapan, a region located between the Sierra Madre Oriental (West) and the coastal plain of the Gulf of Mexico (East) [1]. The study area includes approximately 7551 km^*2*^ (Fig. 2) encompassing 38 municipalities, of which 19 are located in the State of Veracruz and 19 in the State of Puebla [2].

### 2.3 Morphological data acquisition

Nine morphological data traits were measured in the field using the method described by Soto-Arenas and Dressler [3], namely: leaf length (LL) (cm), leaf width (LW) (cm), leaf thickness (LT) (mm), stem diameter (SD) (cm), stem thickness (ST) (cm), distance between nodes (ND) (cm), stem texture degree (STD) (-), flower colour intensity (FC) (-), and fruit length (FL) (cm).

### 2.4 Phytochemical data acquisition

Samples of stems and leaves from every plant were collected and then subjected to a dehydration process that required three days at a temperature of 50 °C. Then 0.5 g of dry matter was dissolved in 5 mL of 80% methanol [4]. The mixture was filtered using Whatman No.1 filter paper. The tests were performed in triplicate and phytochemical parameters were determined as follows: alkaloids in leaves (AL) and stems using the Dragendorff reagent [5]; tannins in leaves (TL) and stems (TS) via a ferric chloride reagent [6]; saponins in leaves (SL) and stems (SS) using a foaming index [7]; phenols in leaves (PL) and stems (PS) by the Folin Ciocalteu reagent [12]; flavonoids in leaves (FL) and stems (FS) using hydrochloric acid and magnesium reagents [8]; and terpenes in leaves (TL) and stems (TS) by the Lieberman test [9]. A thin-layer chromatography technique and corresponding standard of a secondary metabolite were used [10] in order to determine the number of metabolites (Table 5).

### 2.5 Statistical analysis

Data were analysed with a Statistical Analysis System software (v9.0), and ANOVA and Tukey׳s range test (p< 0.05) were later performed. Cluster analysis was conducted using the average data for every variable in order to identify morphological and phytochemical differences between analysed Vanilla species. Subsequently, a principal component analysis was used in order to identify the most influential morphological and phytochemical traits affecting the dispersion of Vanilla species.

### 2.6 Assembly of phylogenetic dendrograms

Subsequently, every phytochemical and morphological data point was hierarchically clustered with the Euclidian Distance Method in order to assemble the phylogenic dendrograms. This procedure was performed with the use of JMP Statistics (v10) [11].
